# Novel role of lncRNA CHRF in cisplatin resistance of ovarian cancer is mediated by miR-10b induced EMT and STAT3 signaling

**DOI:** 10.1038/s41598-020-71153-0

**Published:** 2020-09-08

**Authors:** Wen-Xi Tan, Ge Sun, Meng-Yuan Shangguan, Zhi Gui, Yang Bao, Yu-Feng Li, Zan-Hui Jia

**Affiliations:** 1 Department of Obstetrics & Gynecology, The Second Hospital of Jilin University, 218 Ziqiang Street, Changchun, 130041 Jilin China; 2 Jilin University, Changchun, Jilin China

**Keywords:** Ovarian cancer, Long non-coding RNAs

## Abstract

Ovarian Cancer (OC) is a highly lethal gynecological cancer which often progresses through acquired resistance against the administered therapy. Cisplatin is a common therapeutic for the treatment of OC patients and therefore it is critical to understand the mechanisms of resistance against this drug. We studied a paired cell line consisting of parental and cisplatin resistant (CR) derivative ES2 OC cells, and found a number of dysregulated lncRNAs, with CHRF being the most significantly upregulated lncRNA in CR ES2 cells. The findings corroborated in human patient samples and CHRF was significantly elevated in OC patients with resistant disease. CHRF was also found to be elevated in patients with liver metastasis. miR-10b was found to be mechanistically involved in CHRF mediated cisplatin resistance. It induced resistance in not only ES2 but also OVCAR and SKOV3 OC cells. Induction of epithelial-to-mesenchymal-transition (EMT) and activation of STAT3 signaling were determined to be the mechanisms underlying the CHRF-miR-10b axis-mediated cisplatin resistance. Down-regulation of CHRF reversed EMT, STAT3 activation and the resulting cisplatin resistance, which could be attenuated by miR-10b. The results were also validated in an in vivo cisplatin resistance model wherein CR cells were associated with increased tumor burden, CHRF downregulation associated with decreased tumor burden and miR-10b again attenuated the CHRF downregulation effects. Our results support a novel role of lncRNA CHRF in cisplatin resistance of OC and establish CHRF-miR-10b signaling as a putative therapeutic target for sensitizing resistant OC cells.

## Introduction

Ovarian Cancer (OC) is linked to more deaths than any other cancer affecting female reproductive system and currently ranks fifth among the most deadly cancers affecting women overall^1^. Whereas the incidence of OC is on decline in the US, its constantly on an increase in China, the most populous country in the world^2^. In terms of incidence, OC ranks second behind cervical cancer among the gynecological cancers in Chinese women but in terms of associated mortality as well as the 5-year survival rate, OC has the worst numbers, compared to all gynecological cancers^2^.

Past decades have witnesses growth in our overall understanding of the etiology of OC^3^ which have contributed to the comparative slowing down of incidence, especially in the developed countries. However, the associated mortality remains high, particularly in the developing and under-developed countries. Among the many possible targets for the diagnosis and treatment of OC, the ones involving epigenetic regulation are gaining a lot of interest^4^. Additionally, regulations by miRNAs^5,6^ as well as lncRNAs^7^ are also epigenetic in nature. The reports on miRNAs-mediated tumorigenicity are comparatively more but, of late, there has been increasing interest in the lncRNAs in cancers in general^8–10^ and in OC in particular^11^. Interestingly, the biological action of lncRNAs itself often involves regulation of miRNAs, including in OC^12^.

LncRNAs are now being thoroughly investigated for their role in resistance against chemotherapy in OC in general^13^. Cisplatin has been the chemotherapy of choice against OC for a long time^14^ and the development of resistance against cisplatin remains a major clinical hindrance in the effective therapy of OC patients^15^. Role of several factors^16,17^, including epigenetic factors^18^ has been pursued in the acquired cisplatin resistance in OC. Additionally, in recent years, a number of lncRNAs have been proposed/investigated for their possible role in cisplatin sensitivity and resistance. These lncRNAs include PANDAR^19^ LINC00152^20^, ANRIL^21^ and UCA1^22^ which are increased in cisplatin resistant ovarian cancer cells and thus correlate positively with resistance against cisplatin in ovarian cancers. On the contrary lncRNA ENST00000457645^23^ reverses cisplatin resistance and sensitizes ovarian cancer cells to cisplatin. Additionally, a number of other lncRNAs have been reported in literature to impact cisplatin resistance in different human cancers^24^. Our present study was designed keeping in mind the enormous utility of lncRNAs in OC’s cisplatin resistance. We first evaluated the lncRNAs differentially regulated in cisplatin resistant cells and then in patient-derived specimens. We also performed mechanistic studies to list the mechanism of action of the most promising lncRNA, CHRF and found miR-10b to be the miRNA positively regulated by CHRF. Finally, the results were confirmed in vivo where a role of CHRF, through regulation of miR-10b, was established in cisplatin resistance of OC.

## Materials and methods

### Cell culture

Ovarian cancer cells ES2, OVCAR3 and SKOV3 cells were from ATCC (USA). ES2 and SKOV3 cell lines were cultured in McCoy’s 5a medium whereas OVCAR3 cell line was cultured in RPMI medium with 10% FBS and 1% antibiotics. Cells were cultured in 5% CO_2_ humidified incubator at 37 °C. Cisplatin-resistant ES2 cells were generated by exposing the parental ES2 cells to increasing doses of cisplatin over a period of 5 months with gradual dose escalations every 3 weeks.

### Human specimens

Serum samples were collected at the affiliated hospitals of Jilin University and informed consent was obtained from all subjects. Protocol for this study was approved by the Ethics Committee of Jilin University (JU31775). The first group consisted of 8 patients with cisplatin resistance (tumors and adjacent tissues from the same patient). We also enrolled 6 patients with and 6 patients without liver metastasis. The research team had no information on the identity of research participants. All samples were stored in a − 80 °C freezer.

### RNA preparation

We used RNAiso reagent (TaKaRA, China) to isolate RNA by following the provided instructions. Quality of RNA was checked using a Nanodrop 2000 instrument (ThermoFisher Scientific, China). Samples with OD_260_/OD_280_ > 1.9 were used for further analysis and integrity of RNA was checked using Bioanalyzer (Agilent Technologies, Japan) that utilized an RNA 6000 Nano LabChip.

### Quantitative RT-PCR for detection of lncRNAs and miRNAs

The primers and reagents for the detection of lncRNAs and miRNAs were purchased from Qiagen (China). Only the nuclease-free water was used for all analyses involving these RNAs. RT^2^ first strand kit (Qiagen, China) was used for cDNA synthesis. To 1 μg of RNA, 2 μl of genomic DNA elimination mix was added and mixed, incubated for 5 min at 42 °C and then quickly transferred to ice-cold water for 1 min. Reverse transcription mix (5× buffer with reverse transcriptase enzyme) was then added and incubated for 15 min at 42 °C. Reaction was stopped by heating the mixture to a temperature of 95 °C. All lncRNAs were detected using probes from Qiagen (China). qPCR for miRNAs was conducted using probes and primers from Thermo Scientific Fisher (China) according to manufacturer’s instructions. Results were normalized using glyceraldehyde-3-phosphate dehydrogenase (GAPDH) or U6 as an internal control.

### CHRF downregulation

CHRF downregulation was achieved using locked nucleic acid GapmeR from Qiagen (China). For control conditions, a control LNA GapmeR was used. Cells were transfected at ~ 60% confluency with 20 nM LNA GapmeRs, using Lipofectamine RNAiMax (Thermo Fisher Scientific, China) using recommended protocol.

### miR-10b mimics transfections

miR-10b mimics (GenePharma, China) or the corresponding control miRNA mimics were transfected, using Lipofectamine 2000 transfection reagent (Thermo Fisher Scientific, China), as per the manufacturer’s instructions. Transfections were carried out when the cells were actively dividing and were in logarithmic growth phase.

### Proliferation assay

We evaluated the proliferation of cells, using Cell Proliferation Assay kit (ATCC, USA) which works by measuring the reduction of tetrazolium MTT (3-(4,5-dimethylthiazolyl-2)-2,5-diphenyltetrazolium bromide) by cells that are metabolically active. Cells were counted and 3,000–5,000 cells were seeded in 96 well plates, based on the growth of individual cell lines. Cells were treated with increasing doses of cisplatin, as indicated for individual experiments, for 96 h. At the end of treatment, 10 μl MTT reagent was added for 3 h and, thereafter, 100 μl detergent reagent for 4 h. Quantifications were carried out on a plate reader at 575 nm.

### STAT3 phosphorylation ELISA

We used HTRF^®^ (Homogeneous Time Resolved Fluorescence) cell based phospho and total STAT3 assay kit (Cisbio, China) for quantitation of phosphorylated STAT3. The assay is based on TR-FRET (Time-resolved fluorescence resonance energy transfer) sandwich immunoassay technique. Control, CR cells or the miRNA-transfected/CHRF-silenced OC cells were lysed by using the lysis buffer from the kit for 45 min. Soluble supernatants were collected by centrifugation and the phosphorylated and total STAT3 were quantitated, as suggested in the provided Instructions.

### In vivo cisplatin resistance model

The experiments involving mice were approved by the Animal Welfare Committee of Jilin University (protocol # 19-00150). All methods were performed according to the relevant guidelines and regulations. Female athymic nude mice (Vital River Laboratory Animal Technology Co. Ltd., Beijing, China) were maintained under specific pathogen-free conditions with free access to drinking water and housed in a restricted access room under a 12 h light/ 12 h dark cycle with controlled temperature environment. They were inoculated subcutaneously in the flank with 0.1 ml of cell suspension containing 3.0 × 10^6^ ES2 OC cells. When tumors were measurable, they were measured using calipers, and volume was calculated using the formula: volume = length × width^2^/2. At 7 days time point, a group of mice received ‘pretreatment’ dose (0.75 mg/kg) of cisplatin. All animals received a higher dose (3.0 mg/kg) a week later and the tumor volume measured for following few weeks, as indicated.

### Statistical analysis

Each experiment was repeated at least three times and representative results are shown. Student t (unpaired, one-tailed) was used to compare individual test groups with the respective controls. p value < 0.05 was considered significant.

## Results

### Cisplatin resistance in ES2 cells, and dysregulated lncRNA expression

**Figure 1:**
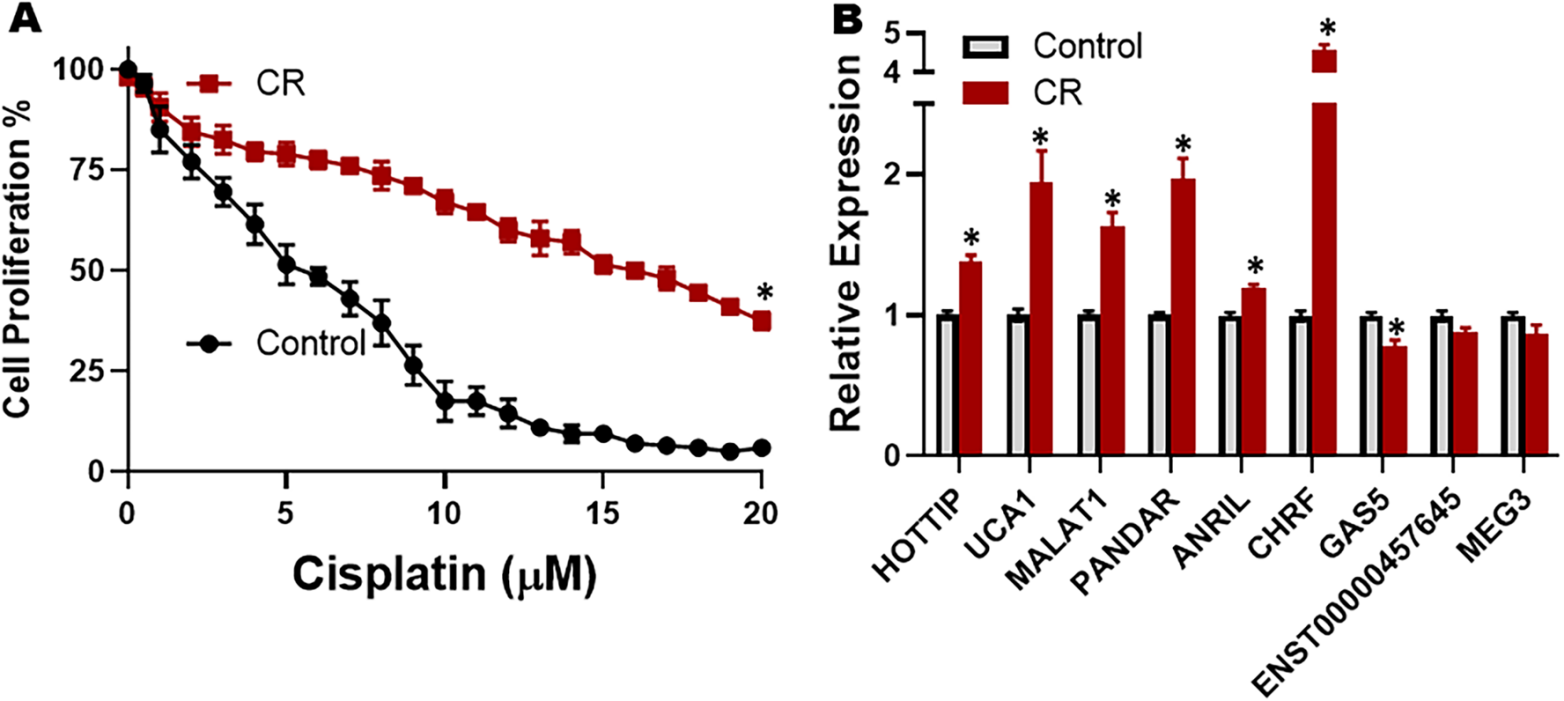
Cisplatin Resistant (CR) ES2 cells and their lncRNA expression profile. (**A**) Proliferation of parental vs CR ES2 cells was measured by MTT assay as described in Methods after their culture in cisplatin containing media (indicated concentrations of cisplatin on X-axis) for 96 h. (**B**) Expression of various lncRNAs in CR ES2 cells as quantitated by quantitative RT-PCR, relative to the parental ES2 cells (control). For each lncRNA, the expression in control parental cells was set to ‘1’ and the relative expression in CR ES2 cells is shown. *p < 0.01, relative to control.

As our first step towards finding an appropriate model for studying the cisplatin resistance in OC cells, we exposed ES2 OC cells to increasing concentrations of cisplatin, as described in “Materials and methods”, to obtain the derivative cisplatin resistant ES2 cells (CR cells). We checked for their cisplatin resistance by exposing them to increasing doses of cisplatin for 96 h followed by evaluation of cisplatin sensitivity by subjecting the cells to MTT assay. The sensitivity of CR cells was compared to that of parental ES2 cells (control). MTT assay revealed a significant (p, 0.01) upregulation of cisplatin resistance in CR cells (Fig. 1A) with IC-50 of CR cells markedly improved, compared to that of control cells. The IC-50 of control cells was 5.1 ± 0.3 μM whereas that of CR cells was 16.4 ± 0.2 μM.

With the goal to evaluate a role of lncRNAs in the cisplatin resistance of OC, we evaluated the levels of different lncRNAs in CR cells, relative to the parental cells. Some of the shortlisted lncRNAs have been reported to affect OC cells’ growth and proliferation while others were chosen for their documented role in human cancers in general. As shown in Fig. 1B, we saw a significant (p < 0.01) change in the expression of several lncRNAs, such as. HOTTIP, UCA1, MALAT1, PANDAR, ANRIL and GAS5. However, the lncRNA with the most change in expression level was determined to be CHRF.

### lncRNAs in cisplatin resistance and liver metastasis of OC patients

**Figure 2:**
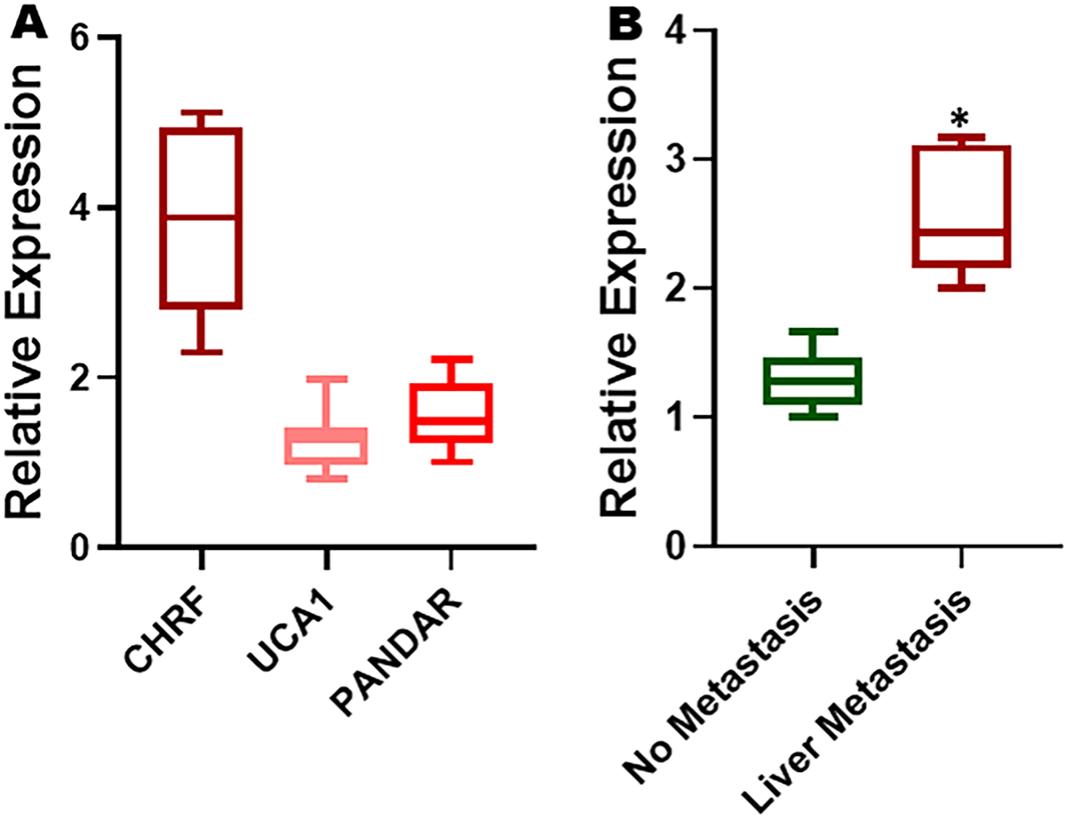
lncRNA validation in human samples. (**A**) The levels of top 3 lncRNAs were quantitated by quantitative RT-PCR in ovarian tumor samples from patients with diagnosed cisplatin resistance (n = 8). The levels shown are relative to levels in adjacent non-tumor tissue. (**B**) Levels of CHRF were evaluated in OC patients with liver metastasis (n = 6) by quantitative RT-PCR and compared to those in OC patients without liver metastasis (n = 6). *p < 0.01, relative to ‘no metastasis’.

After the initial screening in cell line model, and before moving to more detailed mechanistic studies, as described later, we first wanted to evaluate if this data from a paired cell line model was relevant to human OC patients, particularly those with progressed disease. We, therefore, turned to our Hospital repository and looked for tumor samples of OC patients, with particular interest in OC patients who had progressed after cisplatin treatment. We evaluated the levels of top 3 differentially regulated lncRNAs (from Fig. 1B) in the tumor tissues of OC patients with diagnosed cisplatin resistance. The levels in Fig. 2A are relative to expression of same lncRNAs in the adjacent normal tissues. We found that in the tumor tissues as well, CHRF levels were the most up-regulated, compared to UCA1 and PANDAR.

While our focus is on cisplatin resistance of OC and the mechanism involved, we are cognizant of how closely related OC drug resistance and metastasis are^25^. Therefore, we also looked for tumor samples from patients whose OC had metastasized to liver, a common site of OC metastasis^26,27^. We evaluated the levels of CHRF, the lncRNA which was found to be most differentially expressed in CR cells as well as patient samples above. As shown in Fig. 2B, we found that CHRF levels were very significantly upregulated in liver mets of OC patients with liver metastasis, as compared to the levels in ovarian tumor tissues of OC patients with no diagnosed metastasis.

### miRNAs in cisplatin resistance and liver metastasis

**Figure 3:**
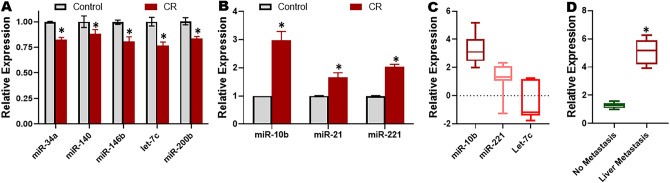
miRNAs in cisplatin resistant cells and human samples. Expression of various (**A**) tumor suppressor and (**B**) oncogenic miRNAs in CR ES2 cells as quantitated by by quantitative RT-PCR, relative to the parental ES2 cells (control). For each miRNA, the expression in control parental cells was set to ‘1’ and the relative expression in CR ES2 cells is shown. *p < 0.01, relative to control. (**C**) The levels of top 3 miRNAs were quantitated in ovarian tumor samples from patients with diagnosed cisplatin resistance (n = 8) by quantitative RT-PCR. The levels shown are relative to levels in adjacent non-tumor tissue. (**D**) Levels of miR-10b were evaluated in OC patients with liver metastasis (n = 6) by quantitative RT-PCR and compared to those in OC patients without liver metastasis (n = 6). *p < 0.01, relative to ‘no metastasis’.

Having established CHRF as the lncRNA that is upregulated in CR cells as well as cisplatin resistance and liver metastasis of human OC patients, we next checked for the levels of different miRNAs in our CR paired cell line model. We scanned the published literature to look for promising miRNAs that have been reported to be involved in cancer drug resistance and/or cancer metastasis. We evaluated the levels of both tumor suppressor miRNAs as well as oncogenic miRNAs and found a few tumor suppressor miRNA (miR-34a, miR-140, miR-146a, let-7c and miR-200b) that were significantly (p < 0.01) downregulated (Fig. 3A). On a similar note, we also found several oncogenic miRNAs (miR-10b, miR-21 and miR-221) to be significantly (p < 0.01) upregulated in CR cells, compared to the parental cells (Fig. 3B). Overall, miR-10b was the most differentially regulated miRNA in CR cells and therefore, we next checked its levels in OC patients’ samples. As shown in Fig. 3C, miR-10b was the most upregulated miRNA in OC patients who were resistant to cisplatin. For this evaluation, we chose the top 3 differentially regulated miRNAs from Fig. 3A,B. miR-10b was further tested in patients with liver metastasis and again levels of miR-10b in liver mets of OC patients were found to be significantly (p < 0.01) elevated, compared to OC patients with no metastasis (Fig. 3D).

### Evaluation of CHRF-miR-10b axis

**Figure 4:**
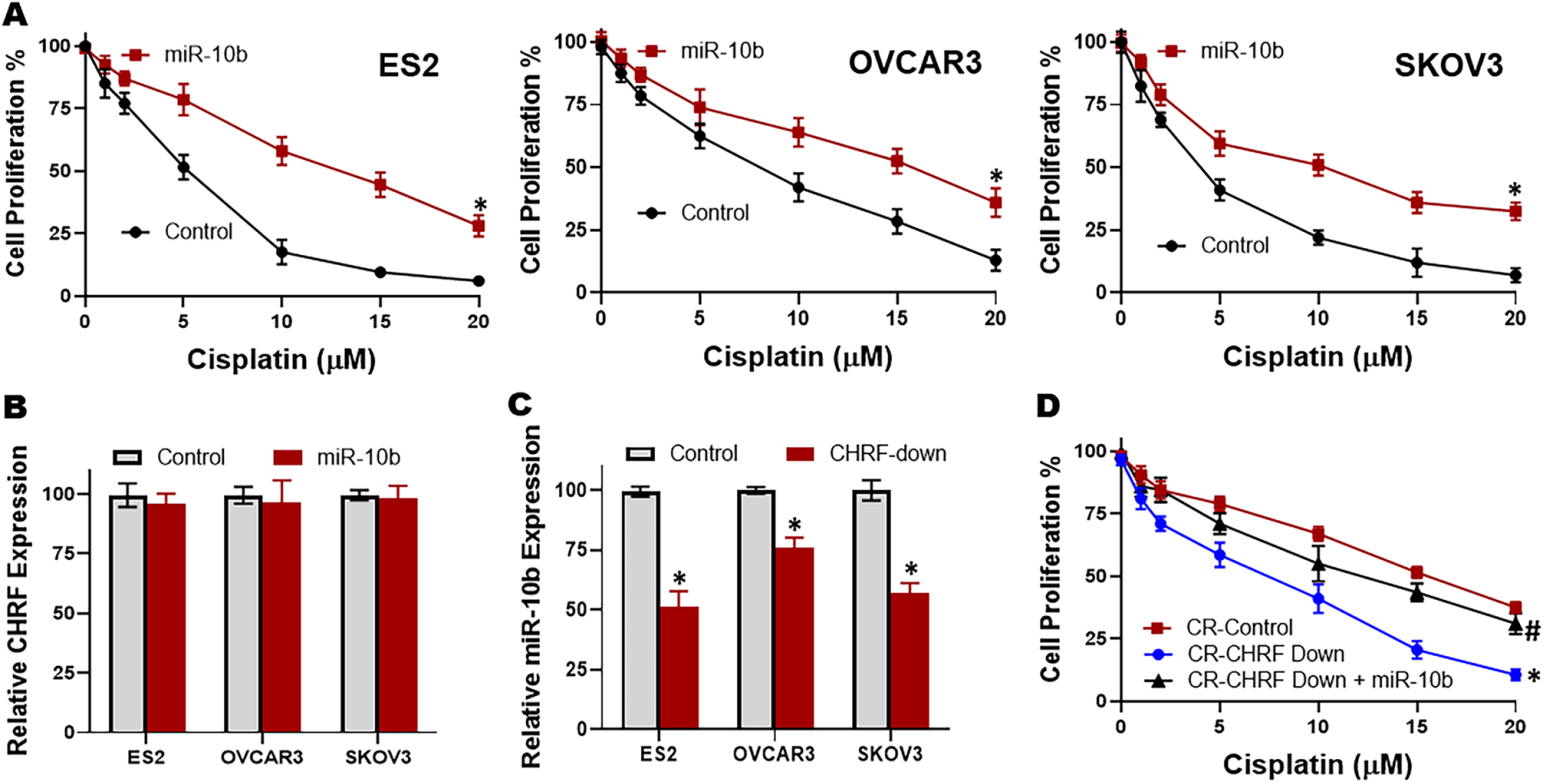
CHRF-miR-10b axis in multiple OC cell lines. (**A**) miR-10b induces cisplatin resistance. miR-10b was transfected in 3 different OC cell lines (ES2, OVCAR3, SKOV3) and effect of increasing doses of cisplatin was evaluated by MTT assay after 96 h of cisplatin treatment. (**B**) Expression of lncRNA CHRF in different OC cells as quantitated by quantitative RT-PCR, relative to the respective parental cells (control). The expression of CHRF in control parental cells was set to ‘1’ and the relative expression in miR-10b transfected cells is shown. (**C**) Expression of miR-10b in different OC cells as quantitated by quantitative RT-PCR, relative to the respective parental cells (control). The expression of miR-10b in control parental cells was set to ‘1’ and the relative expression in cells with down-regulated CHRF is shown. (D) miR-10b attenuates the effects of CHRF down-regulation in cisplatin resistant (CR) ES2 cells. CR ES2 cells (CR-control) and CR ES2 cells with down-regulated CHRF (without and with miR-10b transfections) were subjected to 96 h of cisplatin treatment (increasing doses, as indicated on X-axis) and then the proliferation evaluated by MTT assay. *p < 0.01, relative to control, ^#^p < 0.01, relative to CR-CHRF-Down.

We evaluated whether miR-10b could impact cisplatin resistance. To do this, we transfected pre-miR-10b oligos in ES2 cells and subjected the cells to increasing doses of cisplatin for 96 h. As shown in Fig. 4A, ES2 cells with miR-10b transfections exhibited significantly (p < 0.01) enhanced resistance against cisplatin. In order to rule out cell-specific effects, we performed similar assays in two additional OC cell lines, OVCAR3 and SKOV3. Again, we found similar effects of miR-10b and transfections with this miRNA significantly (p < 0.01) enhanced cisplatin resistance (Fig. 4A).

We next wanted to check the mutual relationship between CHRF and miR-10b. So, we first checked for the expression of CHRF in ES2 cells that were transfected with miR-10b. We found that, relative to control cells, miR-10b transfections did not alter CHRF levels. Atleast, the changes did not reach any statistical significance (Fig. 4B).

In a reciprocal assay, miR-10b levels were significantly (p < 0.01) downregulated upon downregulation of CHRF (Fig. 4C) thus suggesting that miR-10b is positively regulated by CHRF. To further establish such a correlation, we performed another proliferation assay, this time using CR cells as control and comparing their proliferation with CR cells wherein CHRF was downregulated. The cells were exposed to increasing doses of cisplatin for 96 h. As shown in Fig. 4D, proliferation was significantly (p < 0.01) reduced by cisplatin when CHRF was downregulated, thus verifying a role of CHRF in cisplatin resistance of OC cells. Further, when downregulation of CHRF was accompanied by pre-miR-10b transfections, miR-10b could attenuate the effects of CHRF downregulation (Fig. 4D), thus validating a CHRF-miR-10b axis in cisplatin resistance.

### CHRF-miR-10b-mediated cisplatin resistance involves EMT and activation of STAT3

**Figure 5:**
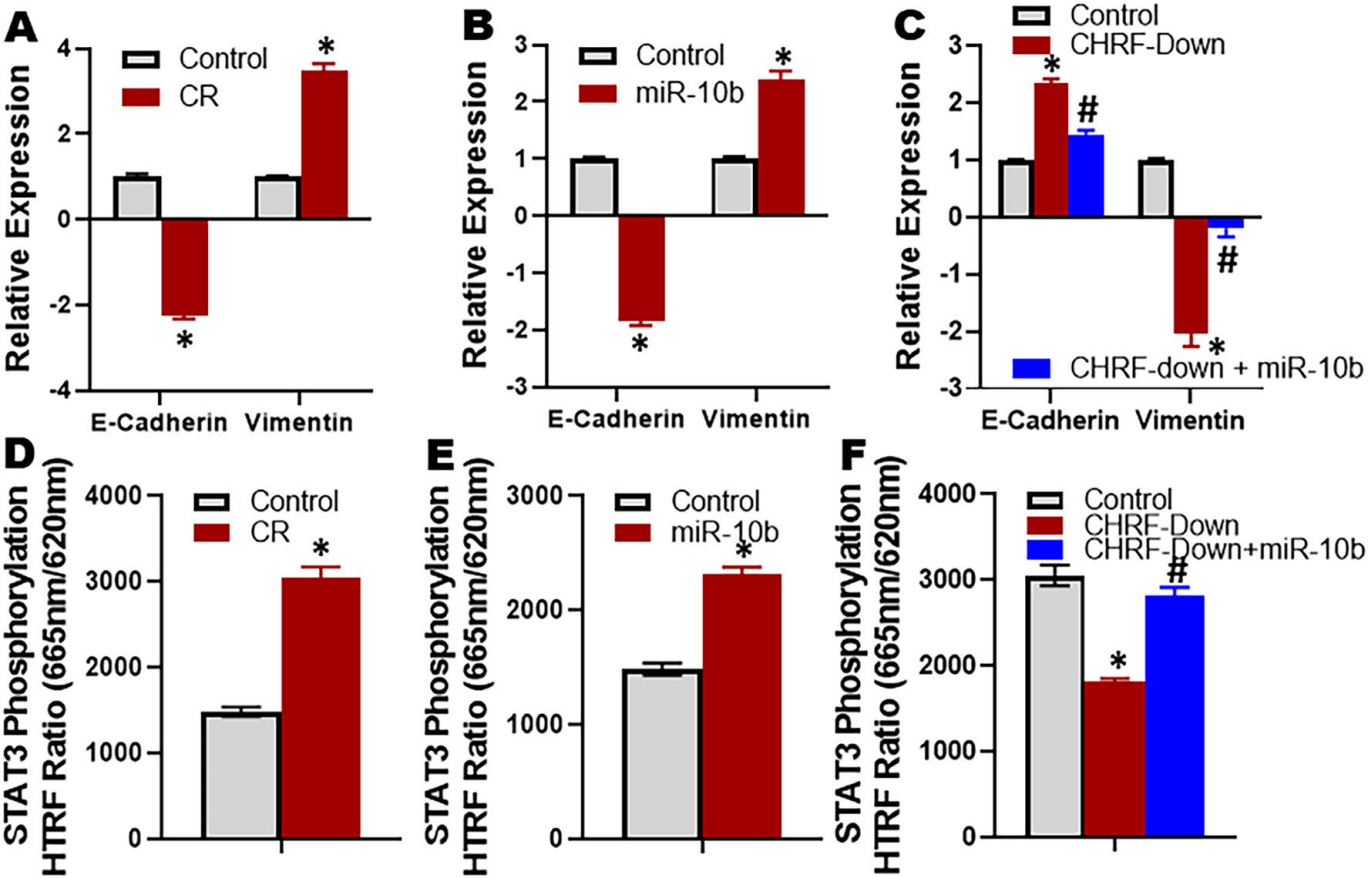
EMT and STAT3 phosphorylation in CR cells and role of CHRF-miR-10b. EMT markers E-cadherin and Vimentin were quantitated using quantitative RT-PCR in (**A**) CR and (**B**) miR-10b transfected ES2 cells, relative to parental cells (control). For each EMT marker, the expression in control parental cells was set to ‘1’ and the relative expression in CR/miR-10b transfected ES2 cells is shown. (**C**) miR-10b attenuates the effects of CHRF down-regulation on EMT markers in cisplatin resistant ES2 cells. CR ES2 cells (control) and CR ES2 cells with down-regulated CHRF (without and with miR-10b transfections: CHRF-Down and CHRF-down + miR-10b, respectively) were subjected to quantitative RT-PCR for the evaluation of EMT markers E-cadherin and Vimentin. The expression levels of EMT markers in control group were set as ‘1’ and the relative expressions in other groups are reported. STAT3 phosphorylation was quantitated using ELISA in (**D**) CR and (**E**) miR-10b transfected ES2 cells, relative to parental cells (control), as described in “Materials and methods” (**F**) miR-10b attenuates the effects of CHRF down-regulation on STAT3 phosphorylation in cisplatin resistant ES2 cells. CR ES2 cells (control) and CR ES2 cells with down-regulated CHRF (without and with miR-10b transfections: CHRF-Down and CHRF-Down + miR-10b, respectively) were subjected to ELISA for the evaluation of STAT3 phosphorylation. *p < 0.01, relative to control, ^#^p < 0.01, relative to CHRF-Down.

With the focus on the mechanism of cisplatin resistance in our model, we next turned to evaluation of a few candidate pathways that are known to influence resistance against chemotherapy. We first evaluated EMT and found that whereas the epithelial marker E-cadherin was significantly (p < 0.01) downregulated in CR cells, the mesenchymal marker vimentin was significantly (p < 0.01) upregulated in CR cells (Fig. 5A). Thus, resistance against cisplatin was marked by induction of EMT. The transfections of miR-10b also induced EMT as E-cadherin was significantly (p < 0.01) downregulated while vimentin was significantly (p < 0.01) upregulated in ES2 cells that had been transfected with pre-miR-10b (Fig. 5B). To find such correlation with CHRF, we downregulated CHRF in CR cells and found a reversal of EMT, i.e. mesenchymal-to-epithelial-transition (MET), as suggested by upregulated E-cadherin and downregulated vimentin (Fig. 5C). However, these effects of CHRF were rescued upon ectopic expression of miR-10b (Fig. 5C) suggesting a role of miR-10b in CHRF mediated regulation of EMT/MET.

We also evaluated STAT3 signaling because of its reported role in cancer drug resistance. STAT3 signaling is often activated leading to resistance against therapy. This was found to be the case in our paired cell line model as we found significantly (p < 0.01) elevated STAT3 signaling, as determined by elevated phospho-STAT3, in CR cells, relative to the parental cells (Fig. 5D). Similarly activated STAT3 signaling was also observed when parental ES2 cells were transfected with pre-miR-10b (Fig. 5E). Further, downregulation of CHRF led to significant (p < 0.01) decrease in STAT3 phosphorylation in CR cells and miR-10b significantly (p < 0.01) attenuated these effects of CHRF downregulation (Fig. 5F).

### In vivo validation in a cisplatin resistance model

**Figure 6:**
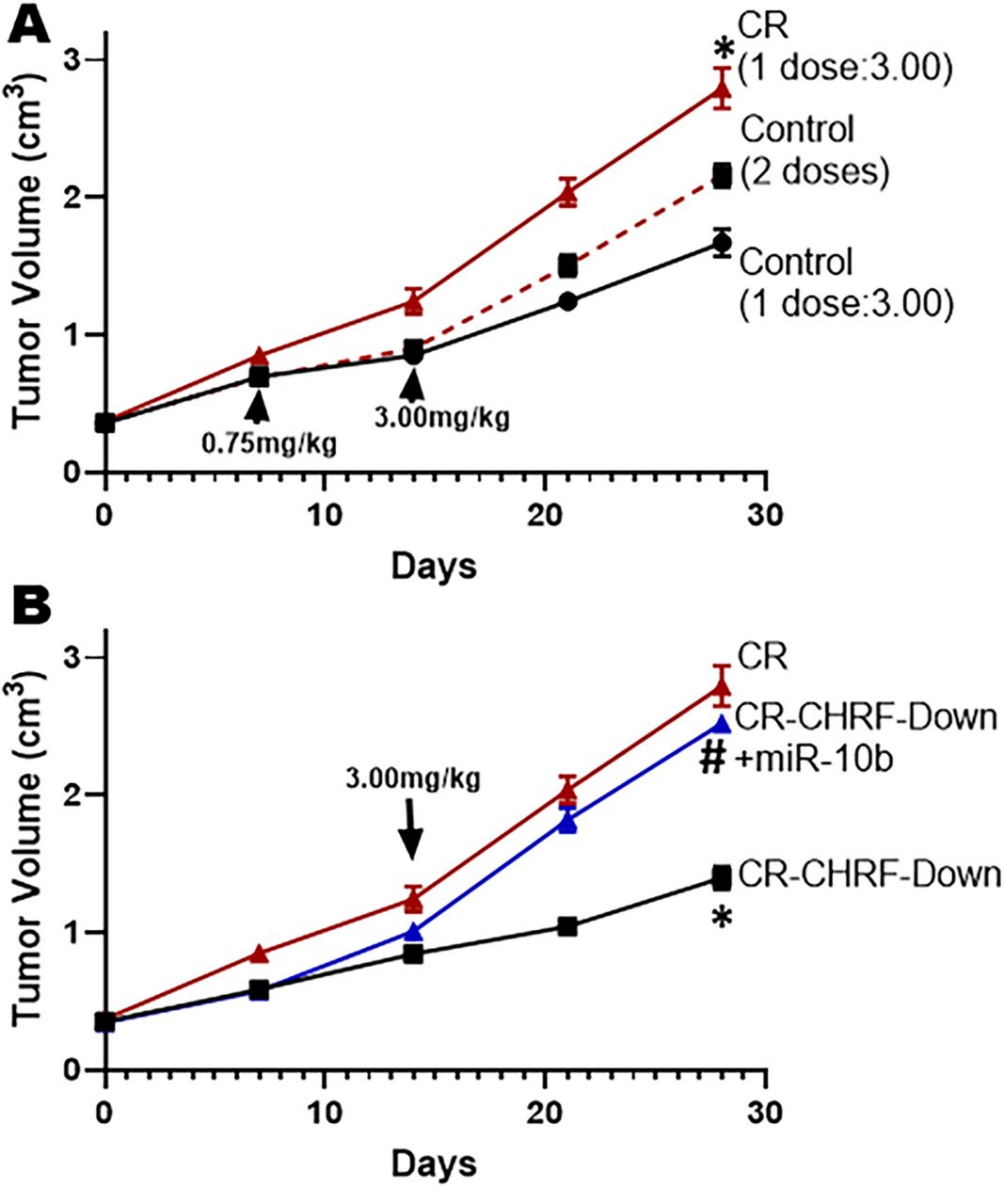
Cisplatin resistance model in vivo and the effects of CHRF-miR-10b axis. (**A**) ES2 cells xenografts were studied in mice in vivo, as described in “Materials and methods”. Mice (n = 10/group) with control ES2 xenografts or CR-ES2 xenografts were challenged with a single dose of 3.0 mg/kg cisplatin at 14 day time point. A control group was administered sub-optimum 0.75 mg/kg dose at 7 day time point to develop resistance against cisplatin. *p < 0.01, relative to control. (**B**) The growth of tumor in CR-ES2 xenografts was further compared to that in CR-ES2 with down-regulated CHRF xenografts (without and with miR-10b: CR-CHRF Down and CR-CHRF-Down + miR-10b, respectively). n = 10 mice per group. *p < 0.01, relative to CR, ^#^p < 0.01, relative to CR-CHRF-Down.

Finally, we performed an in vivo cisplatin resistance experiment utilizing mice, as described before^17,28^. As shown in Fig. 6A, xenografts of control ES2 cells receiving a single dose (high dose—3.0 mg/kg at day 14) are more sensitive to cisplatin compared to xenografts of control ES2 cells that received two doses (a low dose of 0.75 mg/kg at day 7 followed by the high dose of 3.0 mg/kg at day 14). However, the comparatively more resistant control xenografts (with two doses) were still considerably less resistant to cisplatin than the xenografts from CR cells (Fig. 6A). Further, downregulation of CHRF in CR cells resulted in significantly reduced xenograft growth, an effect which was attenuated by ectopic expression of miR-10b in CHRF downregulated CR xenografts (Fig. 6B).

## Discussion

Among the gynecological cancer, OC is extremely lethal. The processes of resistance to therapy and metastasis are particularly relevant in terms of the associated mortality. In this study, we focused on understanding the mechanism of cisplatin resistance of OC cells, with a particular interest in the role of non-coding RNAs. We started our study with the generation of CR cells and tested their cisplatin resistance by comparing their proliferation rate, relative to that of the parental cells. Thus, we generated a cell line pair consisting of parental OC cells and their derivatives which are cisplatin resistant. This model is a good representative of clinical cisplatin resistance because of the way cisplatin resistance is developed: by continuous exposure to cisplatin. Expression of lncRNAs was checked in CR cells and compared to the levels in parental cells with the hypothesis that the lncRNAs with a role to play in cisplatin resistance will be expressed at altered levels in CR cells. Indeed this was found to be the case as the expression of a number of putative lncRNAs was found to be significantly altered in CR cells, relative to parental cells. Amongst these lncRNAs, we chose CHRF because this was the top dysregulated lncRNA in our screening. We also checked for miRNA levels because lncRNAs are known to function through regulating miRNAs^29^. This led to identification of miR-10b as the most differentially regulated miRNA in the CR cells. It is worth mentioning that at the time of designing this study, we were expecting to find a down-regulated miRNA as the most potent target of CHRF. This is because in a majority of reported literature, lncRNAs have been shown to sponge miRNAs leading to their documenting activity. This generally means an inverse correlation between lncRNA and the miRNA it sponges. However, that does not mean that a positive correlation between lncRNA and miRNA has not been documented. There are many such reports^30,31^ and our observation about a positive co-relationship between lncRNA CHRF and the miRNA miR-10b clearly supports such relationship.

In our screening, we identified CHRF as a candidate lncRNA that could affect cisplatin resistance. However, before performing mechanistic studies, we checked if this chosen lncRNA was relevant in actual OC patients. For this, we evaluated its expression in OC patients who had documented development of cisplatin resistance. We found elevated levels of CHRF in tumor tissues of these patients which confirmed our cell line-based results and thus increased confidence in the validity of our findings. We did not restrain ourselves to therapy resistance but even evaluated CHRF levels in OC patients with diagnosed liver metastasis. We observed higher CHRF levels in liver mets of OC patients compared to OC tumor samples from patients who had no diagnosis of metastasis. Combined, these observations clearly indicate a possible role of CHRF in cisplatin resistance as well as liver metastasis of OC. We evaluated liver mets as a proof of principle and did not evaluate CHRF levels in any other metastasis of OC. Our study indicates a possible role of CHRF in liver metastasis but a liver-metastasis-specific role of CHRF cannot be established based on our results. Nor can an involvement of CHRF in metastasis of OC to other organs be ruled out. Only future follow up studies with clearly defined aims can answer such questions. Not just CHRF, but we also evaluated miR-10b in patient samples and observed similar results, which gave us confidence that the positive relationship observed in cell line model is relevant in human OC patients as well. Furthermore, we performed in vivo studies using a cisplatin resistance model to further increase the confidence in our findings and again a role of CHRF-miR-10b was established in cisplatin resistance. Thus, based on our in vitro/in vivo studies and the data from patient samples, we believe that our results are very robust.

In addition to the robust cell line, mice-based and human patients, based studies, we ensured that the results were not cell line specific and we used multiple OC cell lines such as ES2, OVCAR3 and SKOV3 to validate our findings. The similar results obtained in all of the three cell lines are indicative of the reproducibility of our observations and the role of CHRF-miR-10b axis in cisplatin resistance of OC cells. We also worked on elucidating the hierarchy of CHRF-miR-10b regulation. Since both CHRF and miR-10b were found elevated in CR cells and patient samples, we questioned whether CHRF regulated miR-10b or vice versa. To answer this, we designed a reciprocal experiment wherein we altered the expression of one and studied the resulting effect on the other. When we manipulated miR-10b levels, no effect on CHRF was observed. This clearly indicated that miR-10b was downstream of CHRF. This was further proved when we altered CHRF levels in OC cells and observed a significant change in miR-10b levels. Moreover, the change in miR-10b correlated positively with CHRF which further validated the findings from cell line and patient-based samples.

The lncRNA CHRF has been evaluated in cardiovascular diseases^32^ and actually derives its name (cardiac hypertrophy related factor) from such role. However, it has been evaluated in human cancers as well and its role described in tumorigenesis. For example, its has been shown to promote colorectal cancer metastasis^33^. CHRF has also been shown to proliferation and EMT of prostate cancer cells^34^. Interestingly, this study with prostate cancer cells identified the positive modulation of miR-10b by CHRF, similar to what we report here, leading to its effects. The CHRF-miR-10b axis described by us in this study has never been reported, neither in OC nor in the cisplatin resistance, and this signifies the innovative findings from our study. lncRNAs are increasingly being recognized to be involved in progression of human cancers^10^ and even in the cisplatin resistance^13,24^ and our results provide more value to such progress in the field. In our present study, CHRF was chosen from a few shortlisted lncRNAs. As presented in our results, a few other lncRNAs were also found to be differentially expressed. However, CHRF was the most significantly dysregulated under cisplatin resistance conditions. It is possible that the other lncRNAs might also play a role in cisplatin resistance but clearly CHRF stood out at the most promising candidate. Future studies should evaluate mechanism of the other lncRNAs, either alone or in conjunction with CHRF. It is possible that a combination of lncRNAs can be used as signature for the acquired cisplatin resistance in OC.

The miRNA regulated by CHRF, miR-10b, is an interesting miRNA in terms of its role in cancer metastasis as well as resistance against therapy^35–38^. This microRNA has also been reported to play a role in induction of EMT^39,40^. These reports corroborate our findings that support a role of EMT-induction by miR-10b. To the best of our knowledge, no one has reported on the ability of miR-10b to play a role in cisplatin resistance and this is another novel finding reported here. Further, we found elevated STAT3 activation in cisplatin resistant cells. As opposed to miR-10b, a role of STAT3 in cisplatin resistance in human cancers^41^, including OC^42^, has been suggested and we report here a new mechanism of STAT3 activation in cisplatin resistant OC which involves CHRF-miR-10b signaling. The effects of either the lncRNA CHRF or the miRNA miR-10b have never been reported earlier. In conclusion, we present evidence to support the possible therapeutic targeting of CHRF-miR10b axis for the re-sensitization of cisplatin resistant OC patients. Future clinical studies should aim to build up on these findings and evaluate the strategies for targeting these non-coding RNAs in patients.
